# Combination therapy based on dual-target biomimetic nano-delivery system for overcoming cisplatin resistance in hepatocellular carcinoma

**DOI:** 10.1186/s12951-023-01840-3

**Published:** 2023-03-14

**Authors:** Yufen Huang, Qinjie Kou, Yanrong Su, Lu Lu, Xisheng Li, Haiye Jiang, Rong Gui, Rong Huang, Xinmin Nie, Jian Li

**Affiliations:** 1 Department of Laboratory Medicine, Third Xiangya Hospital, Central South University, Changsha, 410013 Hunan China; 2 Department of Blood Transfusion, Third Xiangya Hospital, Central South University, Changsha, 410013 Hunan China; 3 Hunan Engineering Technology Research Center of Optoelectronic Health Detection, Changsha, 410000 Hunan China

**Keywords:** Dual-target, Gene silencing, Chemotherapy, Cisplatin resistance

## Abstract

Strategies to overcome toxicity and drug resistance caused by chemotherapeutic drugs for targeted therapy against hepatocellular carcinoma (HCC) are urgently needed. Previous studies revealed that high oxidored-nitro domain-containing protein 1(NOR1) expression in HCC was associated with cisplatin (DDP) resistance. Herein, a novel dual-targeting nanocarrier system AR-NADR was generated for the treatment of DDP resistance in HCC. The core of the nanocarrier system is the metal–organic frameworks (MOF) modified with nuclear location sequence (NLS), which loading with DDP and NOR1 shRNA (R). The shell is an A54 peptide inserted into the erythrocyte membrane (AR). Our results show that AR-NADR efficiently internalized by tumor cells due to its specific binding to the A54 receptors that are abundantly expressed on the surface of HCC cells and NLS peptide-mediated nuclear entry. Additionally, DDP is more likely to be released due to the degradation of Ag-MOF in the acidic tumor microenvironment. Moreover, by acting as a vector for gene delivery, AR-NADR effectively inhibits tumor drug resistance by suppressing the expression of NOR1, which induces intracellular DDP accumulation and makes cells sensitive to DDP. Finally, the anti-HCC efficacy and mechanisms of AR-NADR were systematically elucidated by a HepG2/DDP cell model as well as a tumor model. Therefore, AR-NADR constitutes a key strategy to achieve excellent gene silencing and antitumor efficacy, which provides effective gene therapy and precise treatment strategies for cisplatin resistance in HCC.

**Graphical Abstract:**

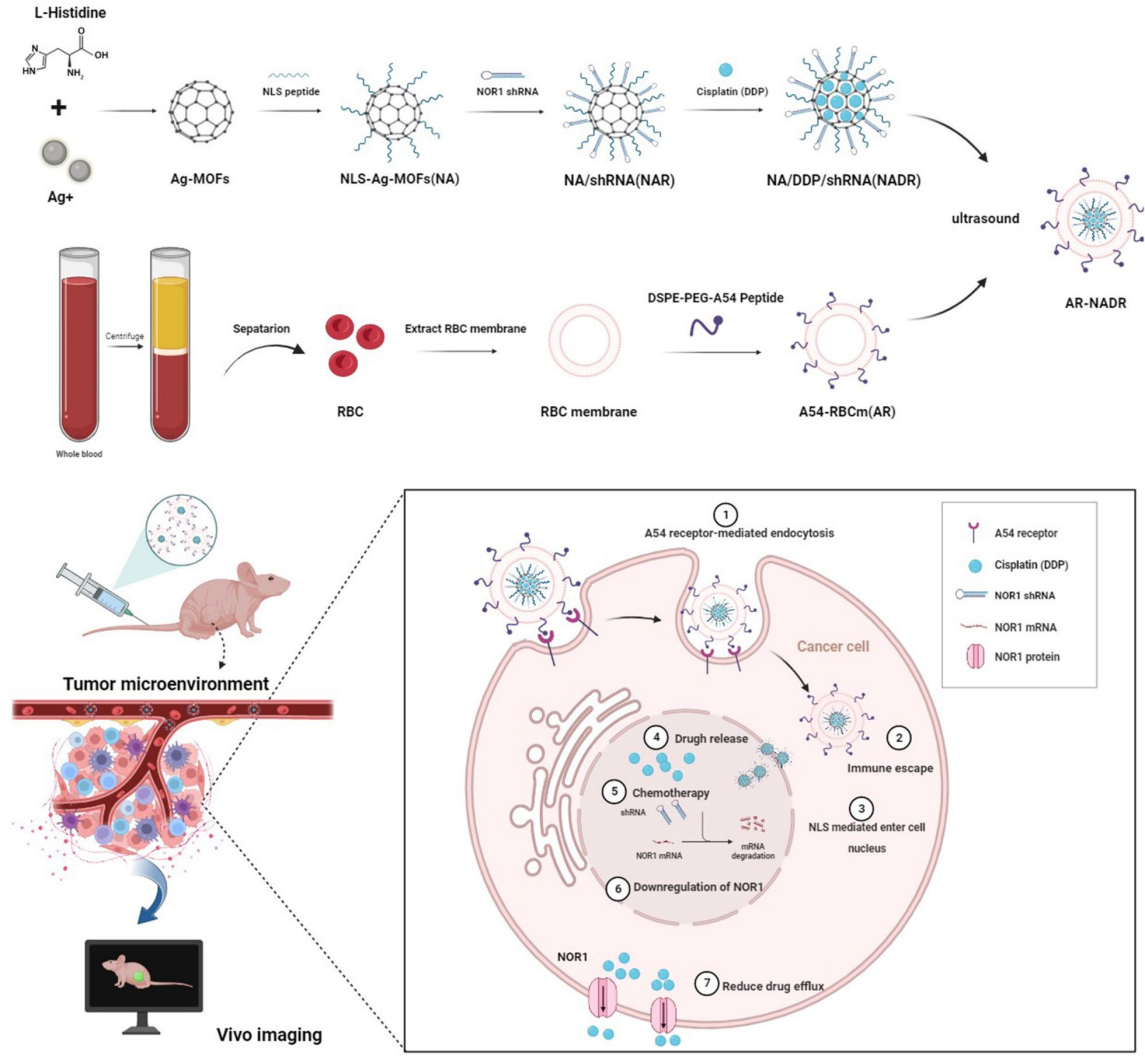

## Introduction

Hepatocellular carcinoma (HCC) is a common malignant tumor characterized by aggressiveness and rapid progression, with high morbidity and mortality [1]. Patients with early HCC are mainly treated by surgery, such as liver resection and liver transplantation. However, 70–80% of patients with HCC are diagnosed at an advanced stage because of tumor progression and unsatisfactory surgical treatments for metastatic lesions. Regional or systemic chemotherapy is a commonly implemented treatment for patients with advanced HCC [2–4]. Cisplatin (DDP) is a classic chemotherapeutic drug for HCC. Unfortunately, the use of DDP in clinical practice is largely limited because severe off-target effects tend to cause a number of treatment-related side effects and serious systemic toxicity [5]. More importantly, multidrug resistance (MDR) causes HCC patients to exhibit a poor drug response, which is the key factor limiting its success [6, 7]. Hence, it is extremely important to develop a valid therapeutic approach to enhance DDP targeting and reverse DDP resistance for HCC therapy.

The oxidored-nitro domain-containing protein 1 (*NOR1*) gene (also called organic solute carrier partner 1, *OSCP1*), first isolated from nasopharyngeal carcinoma (NPC) [8], and located on chromosome 1_P_33.4, is specifically expressed in normal tissues such as the testis, placenta, trachea and brain [9, 10]. Previous studies have revealed that *NOR1* displays moderate to strong expression in HCC [11] and promotes HCC cell proliferation and migration by modulating the Notch signaling pathway [12, 13]. Elevated expression of *NOR1* is related to advanced clinical stage and predicts poor prognosis of HCC [14]. *NOR1* also could activate hepatic stellate cells (HSCs), contributing to liver fibrosis in vitro through the Wnt/β‑catenin signaling pathway [15]. Moreover, NOR1, as a specific organic solute carrier protein, is also widely expressed in the blood-placental barrier, where promotes drug clearances and prevents drugs from entering the fetus through the placenta [16]. It has been reported that methylation of the *NOR1* gene is associated with resistance to imatinib [17]. However, there is little information available regarding its role in the DDP resistance of HCC. Our work revealed that DDP-resistant HCC cells overexpressed *NOR1*. Knocking down *NOR1* expression effectively sensitized DDP-resistant HCC cells to DDP by reducing drug pumping and increasing the accumulation of DDP. Therefore, the *NOR1* gene seems to have emerged as a crucial target for sensitizing HCC cells to chemotherapeutics.

The RNA interference (RNAi) technique is considered a promising therapeutic method for cancer treatment because of its ability to effectively silence genes connected with overexpressed cancer markers and to subsequently resensitize cancer cells [18]. However, gene therapy vectors mainly utilize adenovirus and lentivirus vectors, their potential immunogenicity, carcinogenicity, poor repeatability and high cost hinder widespread clinical application [19–22]. Currently, remarkable progress has been made in the development and application of engineered nanoparticles to treat cancer more effectively [23–26]. Due to their chemical versatility, easy modification, controlled release properties and biosafety, nanocarriers have been widely used to deliver small interfering RNA/short hairpin RNA (siRNA/shRNA) for gene therapy [27–30]. Metal–organic frameworks (MOF) are a class of organic–inorganic hybrid materials consisting of metal ions/clusters and organic ligands, with the advantages of a high specific surface area, porous architecture, and tailorable structure [31]. Recently, biomedical applications of MOF for drug or siRNA/shRNA delivery have attracted close attention [32, 33]. MOF nanocarriers can effectively load drugs and negatively charged genetic materials, which not only protects them from degradation but also accelerates their cellular uptake through the enhanced permeability and retention effect (EPR) effect [34, 35]. In this study, silver ions (Ag^+^) and L-histidine were used to synthesize a porous Ag-MOF that can effectively load chemotherapeutic drugs (DDP) and NOR1 shRNA (R). To enter the nucleus from outside a cell, nanocarriers require special modifications [36]. The surface serialized nuclear localization signal (NLS, amino acid sequence: PKKKRKVG) is considered to be the most classic mediator of nuclear entry [37]. NLS-modified nanoparticles exhibit nuclear targeting by participating in specific intraendosomal processing pathways for endosomal escape without cytotoxicity [36, 38, 39]. Therefore, Ag-MOF modified with NLS will be a feasible method for the efficient delivery of DDP and NOR1 shRNA to the nucleus.

As an exogenous substance, nanomaterials are easily identified and cleared by the mononuclear phagocytic system, resulting in lower blood circulation times [40]. To solve these issues, we propose a novel approach to embellish nanomaterials [41]. Red blood cells (RBCs) are extensively applied in the construction of biomimetic nanomaterials that have a long circulation half-life, incomparable biocompatibility and are easily obtained [42, 43]. In this study, RBC membranes (RBCms) were selected to disguise nanomaterials to help them escape recognition by the immune system and enhance their blood circulation and action time [44]. Ideal nanodelivery systems not only protect the loaded drug from degradation but also possess components that assist the targeting moiety in delivering the drug at the target position [45–47]. The A54 peptide (sequence AGKGTPSLETTP) is an HCC-specific combining peptide containing twelve amino acids selected from a phage display peptide library and effectively localizes to the receptor on the surface of HCC cells [48]. A few studies have revealed that nanomaterials modified with the A54 peptide could specifically target HCC cells, increasing cellular internalization of drug by achieving effective aggregation [49–52]. Conclusively, by inserting the A54 peptide on the surface of RBCms, RBCms are able to bind specifically to the A54 peptide receptor on the surface of HCC cells for increased uptake of nanomaterials.

Consequently, we propose to structure a tumor-targeting, pH-stimulated drug/shRNA codelivery system (AR-NADR) (Fig. 2) that comprises two functional modules, including the core of the nanocarrier system, that is, NLS-modified Ag-MOF (NA) loaded with DDP (D) and NOR1 shRNA (R). The shell is the A54 peptide inserted into RBCm (AR); In particular, AR-NADR can induce a trio of synergetic effects: (i) the dual-targeting effects of the NLS and A54 peptide guide the AR-NADR to the HCC cell nucleus; (ii) the camouflage provided by the RBCms imparts good immune escape ability as well as biocompatibility; and (iii) the downregulation of NOR1 can reverse cisplatin resistance by reducing drug elimination. Thus, this tumor-targeting nanodelivery system is a prospective vector for anticancer drugs and therapeutic RNAi for overcoming drug resistance.

## Materials and methods

### Materials

Silver nitrate (S116266) was purchased from Aladdin (China). Cisplatin (IC0440) and dialysis membrane (2 kDa) (YA1036) were obtained from Solarbio (China). L-histidine (A604351), succinic anhydride (A607835), the MuLV First Strand cDNA Synthesis Kit (B532435) and SYBR Green PCR Mix (B110031) were obtained from Sangon Biotech (China). NLS peptide and A54 peptide were designed by QYAOBIO (China). The shRNA plasmid used to interfere with the *NOR1* gene was designed by GeneChem (China). Polycarbonate porous membrane syringe filters (200 nm) (BS-PES-22) were obtained from Biosharp (China). Penicillin and streptomycin cocktail (PB180120), fetal bovine serum (FBS) (164210-50) and medium (Dulbecco's Modified Eagle Medium (DMEM)) (PM150210) were obtained from Procell (China). TRIzol reagent (15596018) was purchased from Thermo Fisher Scientific (USA). The Cell Counting Kit-8 (CCK-8) (C6005) was manufactured by NCM Biotech (China). Calcein/PI Cell Viability Assay Kit (C2015S), Annexin V-FITC/PI Apoptosis Assay Kit (C1062M), ATP Detection Kit (S0026) and Lipo8000™ Transfection Reagent (C0533) were purchased from Beyotime Biotechnology (China). The Reactive Oxygen Species (ROS) Assay Kit (AKCE002) and Mitochondrial Membrane Potential (MMP) Assay Kit (AKOP013) were purchased from Boxbio (China). The antibody against NOR1 (HPA028436) was manufactured by Sigma-Aldrich (USA). Goat anti-rabbit IgG H&L (HRP) (511203) was purchased from ZENBIO (China).

### Cell culture and selection of mice

HepG2 cells and HepG2/DDP cells were obtained from the Institute for Advanced Study, Central South University (China). The cells were cultured in DMEM containing 10% FBS and 1% penicillin–streptomycin at 37 °C and 5% CO_2_. We chose six-week-old female nude mice and bought the mice from Hunan SJA Laboratory Animal Co., Ltd. (China).

### Transcriptomic analysis

To measure the expression of *NOR1* in DDP-resistant HCC, the total RNA in HepG2 cells and HepG2/DDP cells was extracted using TRIzol reagent. A NanoDrop 2000 (Thermo Fisher Scientific, USA) was applied to measure the concentration and purity of the extracted RNA. Sequencing libraries were generated with a Hieff NGS Ultima Dual-mode mRNA Library Prep Kit for Illumina (12308ES08, Yeasen Biotechnology, China) following the manufacturer’s recommendations. The library quality was assessed on the Agilent Bioanalyzer 2100 system. Subsequently, the samples were sequenced by an Illumina NovaSeq6000. Finally, the raw reads were further processed with a bioinformatics pipeline, namely, the BMKCloud (www.biocloud.net) online platform. Differential expression analysis of the two conditions/groups was performed using DESeq2. Genes with differences in expression levels that exhibited adjusted P values of < 0.05 along with |log2FC|≥ 1.5 were identified as significantly differentially expressed genes. The CCK-8 assay was used to calculate the half-inhibitory concentration (IC_50_) of HepG2 cells and HepG2/DDP cells before/after transfection with NOR1 shRNA.

### Preparation of AR-NADR

#### Construction of Ag-MOF (AM)

L-histidine (0.4 g) and silver nitrate (AgNO_3_, 0.1 g) were added to 5 mL of double distilled water (ddH_2_O). Next, silver nitrate solution was added dropwise to the L-histidine solution. Before the solution changed to milky-white to form Ag-MOF (AM), the solution was placed in a reaction pot at 37 °C and magnetically stirred. Then, the solution was centrifuged at 12,000 rpm for 5 min and washed 3 times with ddH_2_O. The AM powder was dried in a vacuum freeze dryer for storage.

#### Construction of NLS-Ag-MOF (NA)

Ag-MOF (1 mg) was dissolved in 5 mL of ddH_2_O, and succinic anhydride (0.2 mg) was added. The carboxylated Ag-MOF was obtained by centrifugation (12,000 rpm) after magnetic stirring overnight. Carboxylated Ag-MOF (1 mg) was dissolved in 5 mL of ddH_2_O, and then 1-(3-dimethylaminopropyl)-3-ethylcarbodiimide hydrochloride (EDC, 5.3 mg), N-hydroxysuccinimide (NHS, 5.6 mg) and NLS peptide (molecular weight: 940.21, 2 mg) were added and the solution was stirred overnight at 37 °C. Unincorporated EDC, NHS and NLS were filtered by dialysis to obtain NA.

#### Synthesis of NADR (NA/DDP/shRNA)

NA (1 mg) was dissolved in 1 ml of ddH_2_O, PEI (0.1 g) was added to generate positively charge NA, and 50 µg of shRNA plasmid, which was used to interfere with *NOR1* gene expression, was added to react for 2 h with NA/shRNA (NAR). Then, 1 mg of DDP was added, the solution was magnetically stirred at 37 °C in the dark overnight, and NADR was obtained by centrifugation. The absorbances of different concentration gradients of DDP at 301 nm were determined, and the unbound DDP in the supernatant was calculated using standard curves. The calculation formulas for EE and LE were as follows: EE (%) = amounts of DDP loaded on nanocomposite/amounts of DDP added initially × 100. LE (%) = quantity of DDP loaded on the nanocomposite/total quantity of this nanocomposite × 100.

#### Preparation of A54 peptide-inserted RBC membranes (AR)

As mentioned above, the whole blood samples obtained from mice were centrifuged at 3000 rpm for 5 min, and then the supernatant was removed. Finally, the membrane precipitate was washed repeatedly with PBS (3 times) [53]. After washing repeatedly with PBS (0.25X) to release the hemoglobin, the pink erythrocyte membrane precipitate was collected through centrifugation. Finally, nanoscale erythrocyte membrane vesicles were obtained after ultrasound (42 kHz, 100 W) for 5 min and extraction with porous membrane syringe filters (200 nm). In brief, A54 peptide (6.25 mg), DSPE-PEG2000-NH_2_ (5 mg), EDC (2.8 mg), and NHS (1.7 mg) were dissolved in PBS (5 ml) and the solution was magnetically stirred at 37 °C for 24 h. The unbound EDC, NHS and A54 peptide were removed using a dialysis bag. Later, DSPE-PEG2000-NH_2_-A54 (0.1 ml) and the RBC membrane (0.9 ml) were mixed and stirred for 2 h at 37 °C. Eventually, the A54 peptide inserted into the RBC membrane (AR) was synthesized.

#### Construction of A54-RBCm@NLS-Ag-MOF/DDP/shRNA (AR-NADR)

AR was added to an equal volume (0.5 ml) of NADR by ultrasound (5 min, 42 kHz, 100 W). The mixture was then extracted repeatedly 20 times using a porous membrane syringe filter (200 nm). The excess AR in the supernatant was removed through centrifugation (10,000 rpm, 10 min), and the precipitate obtained was the AR-NADR.

### Characterization of AR-NADR

Transmission electron microscopy (TEM; FEI Tecnai F20) was used to confirm the size and morphology of Ag-MOF, NLS-Ag-MOF, NADR, AR, and AR-NADR. The zeta potentials of various nanocomposites were detected using a Zetasizer Nano ZS (Malvern, UK). The X-ray diffraction patterns of Ag-MOF sample are obtained through X-ray diffraction instrument (XRD, Rigaku Ultima IV, Japan). Fourier-transform infrared spectroscopy (FTIR) was used to study the molecular functional groups of Ag-MOF (Thermo Scientific iN10, USA). UV−vis spectrometry (ScanDrop, Analytik Jena, Germany) was used to confirm the construction of AR-NADR. Sodium dodecyl sulfate polyacrylamide gel electrophoresis (SDS‒PAGE) was performed to identify erythrocyte membrane proteins. The gel was stained with Coomassie Blue staining solution (P1300) (SolarBio, China). Finally, the gel imaging system was used for analysis after decolorization.

### Release characteristics of DDP in AR-NADR

To determine whether DDP could be more easily released from AR-NADR once it reached the acidic tumor microenvironment (TME), drug release tests were performed. In short, 1 ml of AR-NADR (the DDP concentration was 1 mg/mL) was placed into a dialysis bag (molecular weight cutoff of 2 kDa) and which was immersed in PBS (20 ml) at pH 7.4 and pH 6.5 separately with magnetic stirring at 37 °C. A 1 mL sample of the dialysate was collected from the incubation medium at specific time points (6, 12, 24, 48, and 72 h), and equal volumes of fresh PBS were placed into the incubation medium. The absorbance of the dialysate was detected at 301 nm (DDP characteristic peak) to measure the concentration of DDP released from the AR-NADR using standard curves.

### Biocompatibility of AR-NADR in vitro

#### Immune escape assay

The immune evasion of AR-NADR was assessed in a RAW264.7 cell uptake assay. RAW264.7 cells were incubated with Cy5.5-labeled NADR, R-NADR and AR-NADR (20 µL) for 6 h and later stained with DAPI. Finally, an inverted fluorescence microscope (Zen2, Zeiss, Germany) was used for imaging.

#### Cytotoxicity assay

The cytotoxicity of AR-NADR was determined using a CCK-8 in Human Umbilical Vein Endothelial Cells(HUVECs). In short, the cells were seeded in 96-well plates (5 × 10^3^ cells/well) for 24 h. Various concentrations of NADR, R-NADR and AR-NADR (0–100 µg/mL) were added and incubated for 24 h. Then, 10 µL of CCK-8 solution was added to each well and incubated for 2 h, and the absorbance was measured at 450 nm.

#### Hemocompatibility

Hemocompatibility was detected with a hemolysis assay. Various concentrations of AR-NADR (0–100 µg/mL) were incubated with 5% red blood cells at 37 °C for 2 h. The supernatant was collected after centrifugation (1500 rpm, 10 min), and a microwell plate detector (EnSpire 2300, PerkinElmer, USA) was applied to detect the absorbance at 545 nm. The positive and negative controls were ddH_2_O and normal saline, respectively. The ratio of hemolysis (%) = (absorbance of experimental sample−absorbance of negative control)/(absorbance of positive control−absorbance of negative control) × 100.

### Double target binding ability of AR-NADR

The targeting effect of the A54 peptide on different tumor cells was detected. First, HepG2 cells (A54 + cells) were treated with 20 μL of RBCm@NLS-Ag-MOFs (RBC-NA) or A54-RBCm@NLS-Ag-MOFs (AR-NA) for 12 h. In addition, the A54 peptide competition test was used for further verification. In brief, the A54 peptide (20 μg) was pretreated for 2 h to block the HepG2 cell surface receptors, and then the cells were treated with 20 μL of AR-NA for 12 h [54]. Additionally, 4T1, A549 and HeLa cells (A54- cells) were treated with 20 μL of AR-NA to confirm the specific targeting of the A54 peptide to HCC cells. The Ag-MOFs were labeled with FITC. The fluorescence of AR-NA in the cells was observed with a fluorescence microscope (Zen2, Zeiss, Germany). Moreover, to detect the effect of the nuclear targeting of the NLS peptide, after 2, 6, and 24 h of incubation, HepG2 cells were exposed to 20 μL of AR@Ag-MOFs (AR-AM) and AR@NLS-Ag-MOFs (AR-NA) for 2 h, 6 h, and 24 h. The Ag-MOFs were labeled with Cy5.5. The cells were then washed with PBS, fixed with 4% paraformaldehyde, and stained with DAPI. The fluorescence was observed by a fluorescence microscope (Zen2, Zeiss, Germany).

### *NOR1* gene silencing/knockdown efficiency, IC_50_ and cell efflux assessments

Agarose gel electrophoresis was carried out to estimate NOR1 shRNA loading in NA. Different NA/shRNA weight ratios (w/w) (the weight of shRNA was 5 µg) were applied to an agarose gel (3%, 100 V) in TAE buffer containing YeaRed Nucleic Acid Gel Stain (10202ES76, Yeasen, China). A UV transilluminator and a digital imaging system (Life Science Technologies, USA) were applied to obtain images. The *NOR1* gene silencing efficiency was sequentially evaluated. HepG2 cells and HepG2/DDP cells were cultured in 6-well plates (5 × 10^5^ cells/well) for 24 h. Later, HepG2/DDP cells were treated for 48 h with PBS, free shRNA, Lipo8000-NC (Lipo8000-negative control plasmid), Lipo8000-shRNA and AR-NAR (AR-NA/shRNA), in which the polyplexes contained the NOR1 shRNA plasmid at a shRNA concentration of 2.5 µg well^−1^. The GFP of the plasmid could emit green fluorescence, which was used to evaluate transfection efficiency by an inverted fluorescence microscope (Zen2, Zeiss, Germany). In addition, total RNA was extracted from cultured cells with TRIzol reagent and reverse transcribed into cDNA with a MuLV First Strand cDNA Synthesis Kit. Then, real-time quantitative PCR (RT‒qPCR) was implemented with a SYBR Green PCR Kit, and the fluorescence was measured using a LightCycler 96 (Roche, USA). All samples were analyzed in triplicate and normalized to GAPDH levels. The relative fold variation in mRNA expression (normalized) was tested on the basis of the 2^−ΔΔCt^ method. The sequences are provided as follows. For HS-GAPDH, the forward primer was CAGGAGGCATTGCTGATGAT; the reverse primer was: GAAGGCTGGGGCTCATTT; For NOR1, the forward primer was: CACTCCTCATCTTCTTCCAA; the reverse primer was: CCTCTTCTTCTTCTTCACCTT. In addition, the mRNA obtained from cells was evaluated by agarose gel electrophoresis (3% agarose gel, 100 V, 20 min). Furthermore, the protein obtained from cells was extracted with RIPA buffer and ultrasound, and the protein concentration was measured with a BCA protein assay kit. Following the Western blotting steps, we detected the expression of NOR1 in the cells with the corresponding antibody. ImageJ software was used for semiquantitative analysis of the agarose gel electrophoresis bands and Western blot bands. To demonstrate that reducing NOR1 expression could sensitize HepG2/DDP cells to DDP, the IC_50_ was calculated by the CCK-8 assay carried out using samples subjected to the above treatments. Furthermore, the cell efflux of DDP at 4, 6, 12, and 24 h was detected. HepG2 cells, HepG2/DDP cells and HepG2/DDP cells (treated with Lipo8000-shRNA) were seeded in 12-well plates (5 × 10^4^ cells/well) for 24 h. Then, DDP (15 μg/mL) was added. After culturing for 4, 6, 12, and 24 h, the medium supernatant was collected at the indicated time points, and the absorbance was measured. The rate of cell efflux of DDP was calculated with a standard curve.

### Antitumor action of AR-NADR in vitro

First, the toxicity of AR-NADR was determined by the CCK-8 test. HepG2 cells and HepG2/DDP cells were cultured in 96-well plates (5 × 10^3^ cells/well) for 24 h. Then, the cells were exposed to AR-NA, AR-NAR, DDP, AR-NAD, or AR-NADR (the concentration of DDP was 0–40 μg/mL) for 24 h; subsequently, CCK-8 solution (10 µL) was added to each well for incubation at 37 °C for 2 h. The relative cell viability was calculated according to the absorbance at 450 nm. Additionally, the IC_50_ was calculated in cells treated with DDP, AR-NAD and AR-NADR. Moreover, live/dead cell staining was performed to estimate the antitumor effects of AR-NADR in vitro. HepG2 cells and HepG2/DDP cells were cultured in 6-well plates (5 × 10^5^ cells/well). Next, the cells were exposed to PBS, AR-NA, AR-NAR, DDP, AR-NAD, or AR-NADR (the concentration of DDP was 15 μg/mL) for 6 h. After the medium was removed, calcein-AM and PI were used for live/dead staining, and the cells were observed with an inverted fluorescence microscope (Zen2, Zeiss, Germany).

### Detection of MMP, ATP, ROS, and apoptosis in vitro

To investigate the mechanism by which AR-NADR overcomes drug resistance, HepG2/DDP cells were inoculated in 6-well plates (5 × 10^5^ cells/well) for 24 h and later exposed to PBS, AR-NA, AR-NAR, DDP, AR-NAD and AR-NADR (the concentration of DDP was 15 μg/mL) for 12 h. Subsequently, the cells were collected. The JC-1 Assay Kit was used to measure the mitochondrial membrane potential (MMP) of cells detected by flow cytometry (Cytek Athena, USA). The intracellular adenosine triphosphate (ATP) and reactive oxygen species (ROS) level were detected with ATP quantification kit and ROS assay kit respectively. An Annexin V-FITC apoptosis detection kit was used to evaluate cell apoptosis by flow cytometry to comprehensively reveal the antitumor effect of AR-NADR in vitro.

### Xenograft tumor models and assay of AR-NADR biodistribution

Six-week-old female nude mice were obtained from Hunan SJA Laboratory Animal Co., Ltd. (China). All the protocols for the proposed in vivo experiments were approved by the Animal Use and Care Committee of Central South University. HepG2/DDP cells (5 × 10^6^ cells/100 μL) were subcutaneously used to inoculate nude mice to prepare a DDP-resistant hepatocellular carcinoma model. The tumor volume was measured using the following formula: volume = length × width^2^/2. The mice were deemed ready for further therapeutic study after the tumor volume reached 100 mm^3^. To evaluate the target of AR-NADR in vivo, 100 μL each of Cy5.5-NADR, Cy5.5-R-NADR and Cy5.5-AR-NADR (DDP, 2 mg/ml) was injected into HepG2/DDP tumor-bearing mice via the tail vein. The fluorescence signals of Cy5.5 from the mice, extracted organs and tumors were further detected with an In Vivo Imaging System (PerkinElmer, USA) at 6, 24, and 48 h after administration.

### Antitumor action of AR-NADR in vivo

HepG2/DDP tumor-bearing mice were randomly divided into six groups (n = 5 per group) and intravenously injected with PBS, AR-NA, AR-NAR, DDP, AR-NAD, or AR-NADR in the tail vein 5 times (2 mg/ml DDP, every 2 days). The tumor size and body weight of the mice were recorded every second day. At 14 days, all the animals were euthanized, and the major organs (heart, liver, spleen, lung, and kidney), tumors and blood samples were collected for histological analysis (hematoxylin–eosin (H&E) staining) and blood tests. Western blotting and immunohistochemical analyses (IHC) were also performed to measure NOR1 expression in tumor tissues. Apoptosis in tumor tissues was detected via terminal deoxynucleotidyl transferase dUTP nick-end labeling (TUNEL) assays based on the standard protocol.

### Statistical analysis

Data are expressed as the mean ± standard deviation (SD) after at least three independent experiments and were assessed by GraphPad Prism software. Intergroup differences were assessed with one-way analysis of variance (ANOVA) and Tukey’s post hoc test. (*p < 0.05, **p < 0.01, ***p < 0.001 and ****p < 0.0001).

## Results and discussion

### Transcriptomic study to explore the effect of *NOR1* on cisplatin-resistant HCC cells

**Fig. 1:**
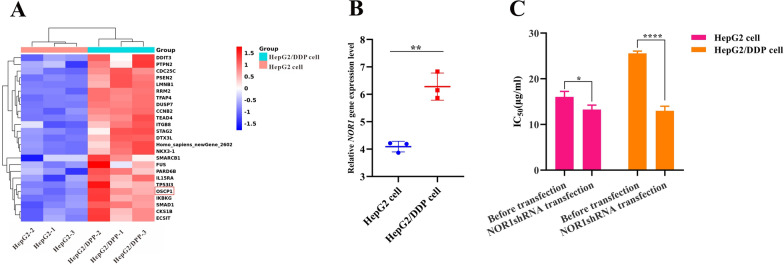
Transcriptomic analysis of HepG2 cells and HepG2/DDP cells. **A** Heatmap show the expression of DEGs between HepG2 cells and HepG2/DDP cells. Red indicates upregulation, and blue indicates downregulation. **B** The relative expression of the *NOR1* gene in HepG2 cells and HepG2/DDP cells. **C** The IC_50_ in HepG2 cells and HepG2/DDP cells before/after transfection with NOR1 shRNA

To evaluate the expression of *NOR1* under conditions of DDP resistance, we conducted transcriptomic analysis on both HepG2 cells (n = 3) and HepG2/DDP cells (n = 3). As shown in Fig. 1A and B, among the differentially expressed genes (DEGs), the expression of the *NOR1 (OSCP1)* gene was significantly increased in the HepG2/DDP groups. After transfection with NOR1 shRNA, the IC_50_ in HepG2/DDP cells was significantly lower than that in HepG2 cells, indicating that the overexpression of the *NOR1* gene was probably related to DDP resistance in HepG2/DDP cells (Fig. 1C). Based on the above findings, overexpressed *NOR1* may affect DDP resistance and could serve as a new target for reversing DDP resistance in HCC.

### Fabrication and characterization of AR-NADR

**Fig. 2:**
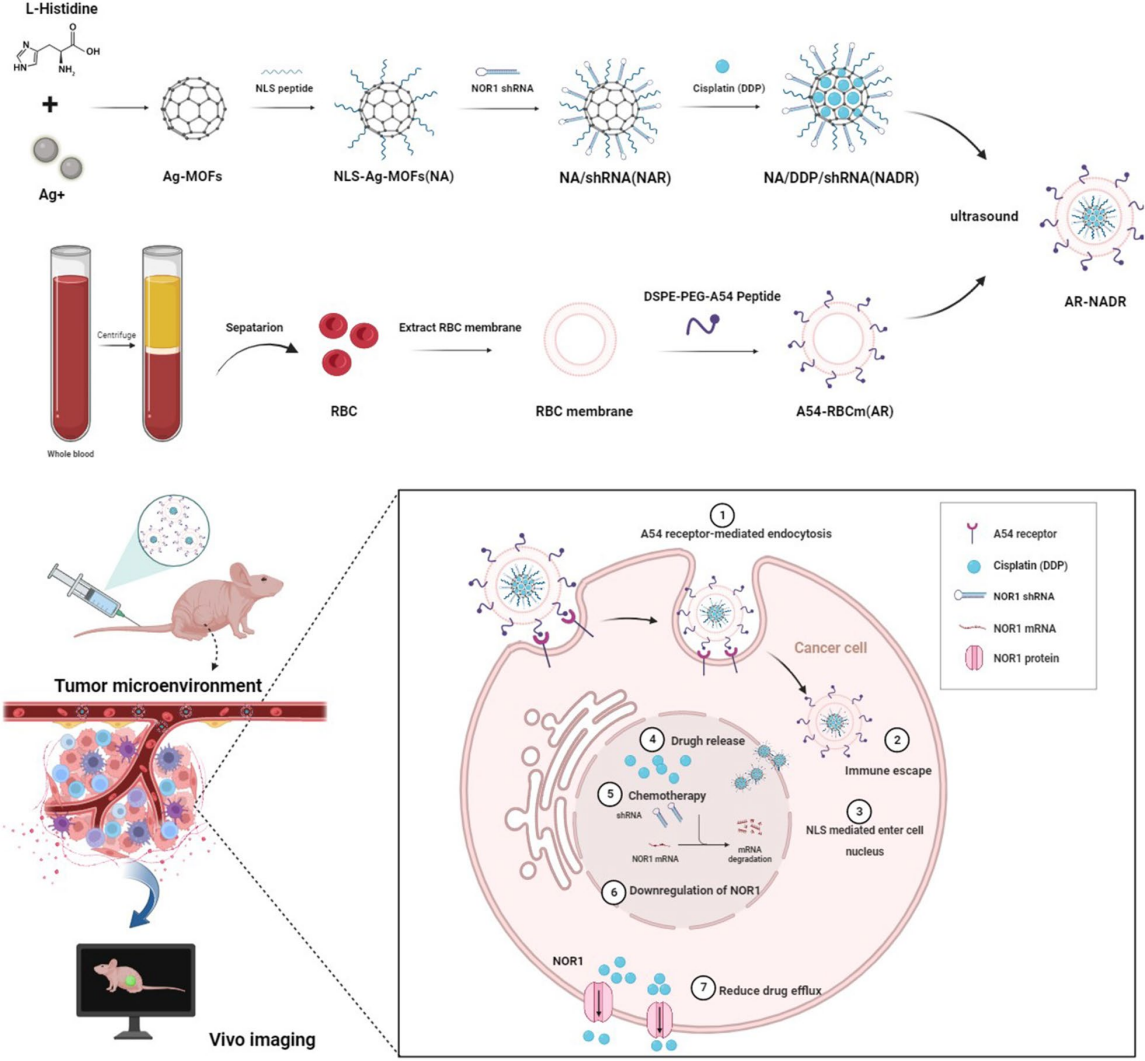
Schematic diagram of A54-RBCm@NLS-Ag-MOF/DDP/NOR1shRNA (AR-NADR) construction and its combination therapeutic mechanisms in the treatment of cisplatin resistance in HCC by interfering with NOR1. **1** A54 receptor-mediated endocytosis. **2** Immune escape. **3** NLS mediated enter into the cell nucleus. **4** Drug release. **5** Chemotherapy. **6** Downregulation of NOR1. **7** Reduced drug efflux

As indicated in Fig. 2, the fabrication of AR-NADR primarily included two processes: I) the construction of an NLS-modified Ag-MOF (NA) synthesized by Ag^+^ and L-histidine loaded with DDP (D) and NOR1 shRNA (R) to constitute a NADR nanocore, and II) the shell, in which the A54 peptide is inserted into a red blood cell membrane vesicle (AR) and is wrapped around the NADR to construct the whole core–shell structure of AR-NADR.

**Fig. 3:**
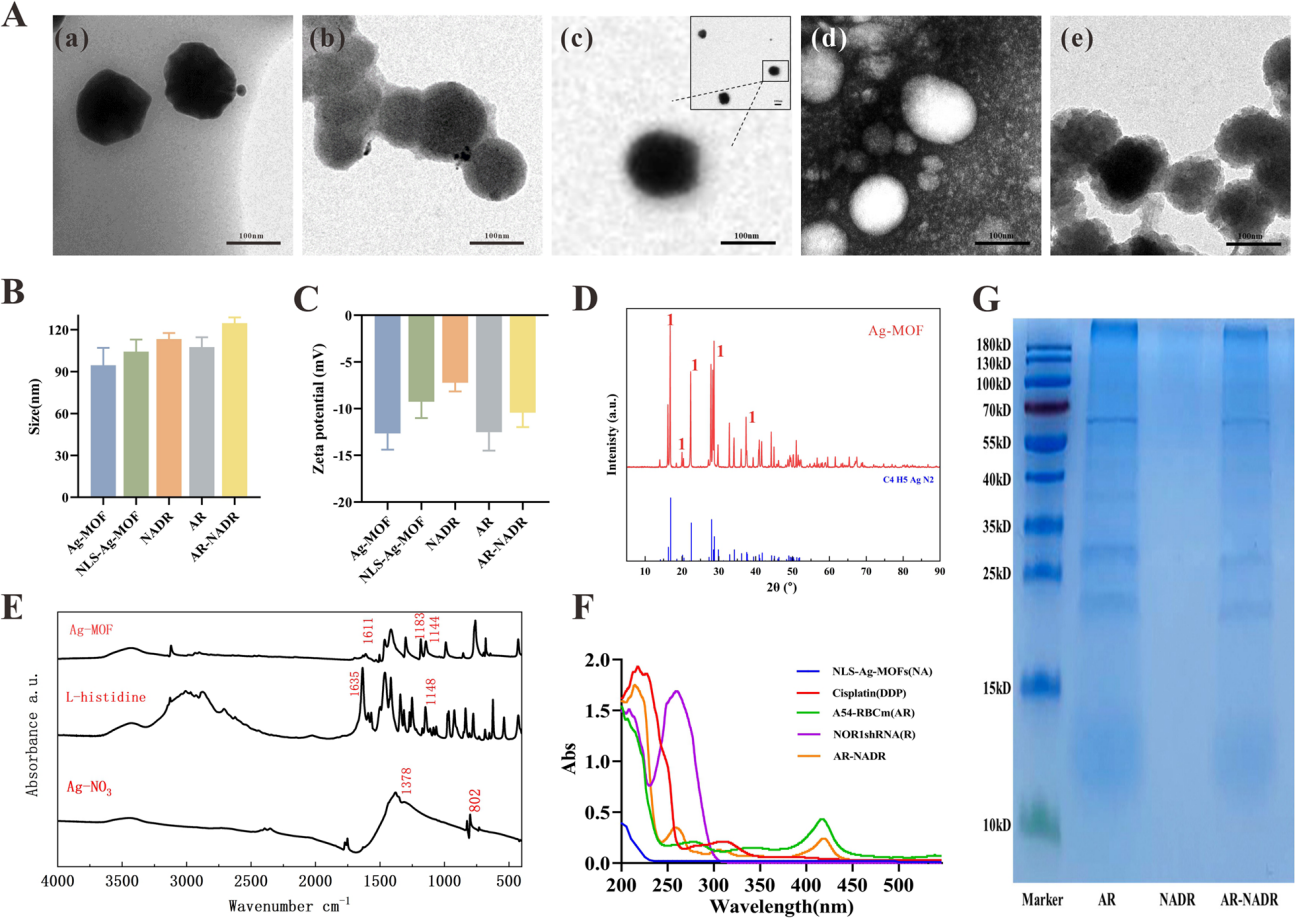
Characterization of AR-NADR. **A** TEM images of (a) Ag-MOF (b) NLS-Ag-MOF (c) NADR, (d) AR and (e) AR-NADR (Scale bar:100 nm). **B** Nanoparticle size and **C** zeta potential of Ag-MOF, NLS-Ag-MOF, NADR, AR, and AR-NADR. **D** XRD patterns of Ag-MOF. **E** FTIR spectra of AgNO_3_, L- histidine and Ag-MOF. **F** UV–vis spectra of NLS-Ag-MOF (NA), Cisplatin (DDP), A54-RBCm (AR), NOR1shRNA (R) and AR-NADR. **G** Profiles of membrane proteins in AR, NADR and AR-NADR by SDS-PAGE analysis. Data are presented as means ± SD (n = 3)

The morphology and size of different nanocomponents were visualized with TEM. As shown in Fig. 3A and B, the size and shape of Ag-MOF change little after modified with NLS. NADR exhibited a uniform spherical morphology with a size of ~ 113 nm (analyzed by the TEM). After decoration with AR, the average particle size of AR-NADR was 124.6 ± 4.04 nm. There was an 11-nm increase in the nanoparticle size, probably due to the membrane coatings, because the average thickness of RBC bilayers is 7–12 nm [55]. In Fig. 3C, the zeta potential of Ag-MOF, NLS- Ag-MOF and NADR were − 12.6 ± 1.7, − 9.2 ± 1.7 and − 7.2 ± 0.9 mV respectively; after fusion with AR, the zeta potential of AR-NADR decreased to − 10.4 ± 1.5 mV. This change might have resulted in AR encapsulation causing a charge shielding effect, which further confirmed the successful encapsulation of AR-NADR. As shown in Fig. 3D, the Ag-MOF were characterized by XRD, the phase detected of Ag-MOF was correspond to Ag (CHN), in which the 2θ of Ag (CHN) was 16.2, 20.02, 22.359, 28.699, and 37.283. Meanwhile, the FTIR spectra results (Fig. 3E) showed the absorption peaks of Ag-MOF all shifted to different degrees. C = O stretching vibration in the histidine was shifted from 1635 cm^−1^ to 1611 cm^−1^, and C–N stretching vibration was shifted from 1148 cm^−1^ to 1183 cm^−1^ and 1144 cm^−1^. This indicated that Ag^+^ in AgNO_3_ reacted with C–N in imidazole ring of histidine to form Ag–N chemical bond, which changed the infrared spectrum absorption peak of histidine. In the UV‒vis spectrum (Fig. 3F), AR-NADR had absorption peaks at 260, 301, and 410 nm, corresponding to the characteristic absorption peaks of NOR1 shRNA, DDP, and AR vesicles, respectively. Moreover, the SDS-PAGE results shown in Fig. 3G indicate that nearly all erythrocyte membrane protein profiles were retained in AR-NADR. All of the above findings further verified the successful preparation of AR-NADR.

### Drug loading and release rate of DDP in AR-NADR

**Fig. 4:**
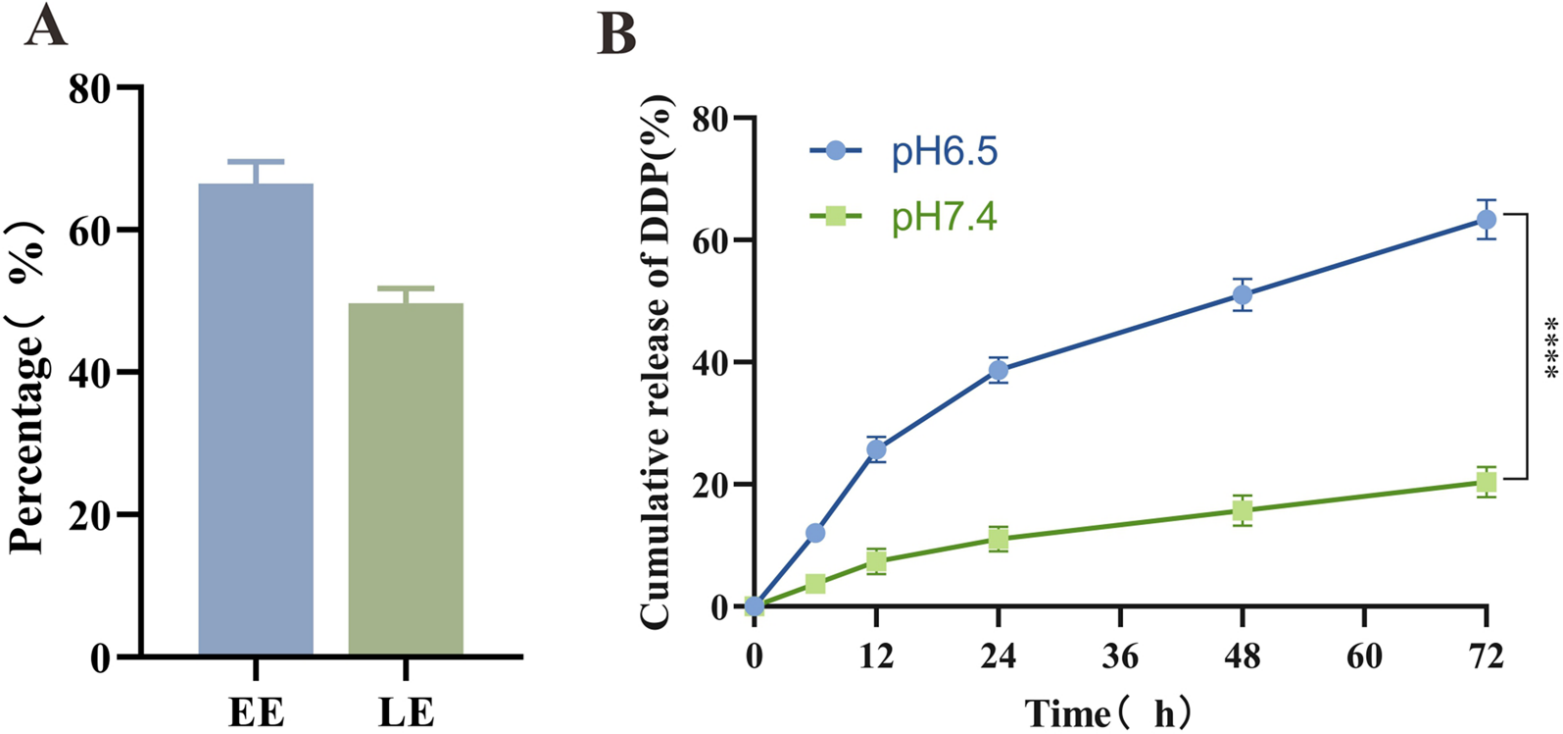
Drug loading and release rate of DDP in AR-NADR. **A** EE and LE of DDP in AR-NADR. **B** Cumulative release rate of DDP from AR-NADR at different pH values (6.5, 7.4). Data are presented as the means ± SD (n = 3) (intergroup comparisons: ****p < 0.0001)

As a metal–organic material, NA synthesized with Ag^+^ and L-histidine served as an ideal carrier for drug/shRNA due to its high specific surface area and porosity. As shown in Fig. 4A, the drug encapsulation efficiency (EE%) and loading efficiency (LE%) of DDP in AR-NADR were 66.5 ± 3.09% and 49.8 ± 2.3%, respectively. Furthermore, the DDP release profiles of AR-NADR were evaluated at pH 7.4 and pH 6.5, which simulated a neutral circulation environment and an acidic tumor microenvironment, respectively (Fig. 4B). Increased cumulative amounts of the released DDP were obtained at pH 6.5 because of accelerated degradation of Ag-MOF in acidic conditions [56]. The release characteristic of the pH response of AR-NADR was especially helpful for drug delivery in a weakly acidic tumor environment and beneficial for enhancing antitumor effects and reducing toxic side effects.

### Immune evasion and biocompatibility of AR-NADR in vitro

**Fig. 5:**
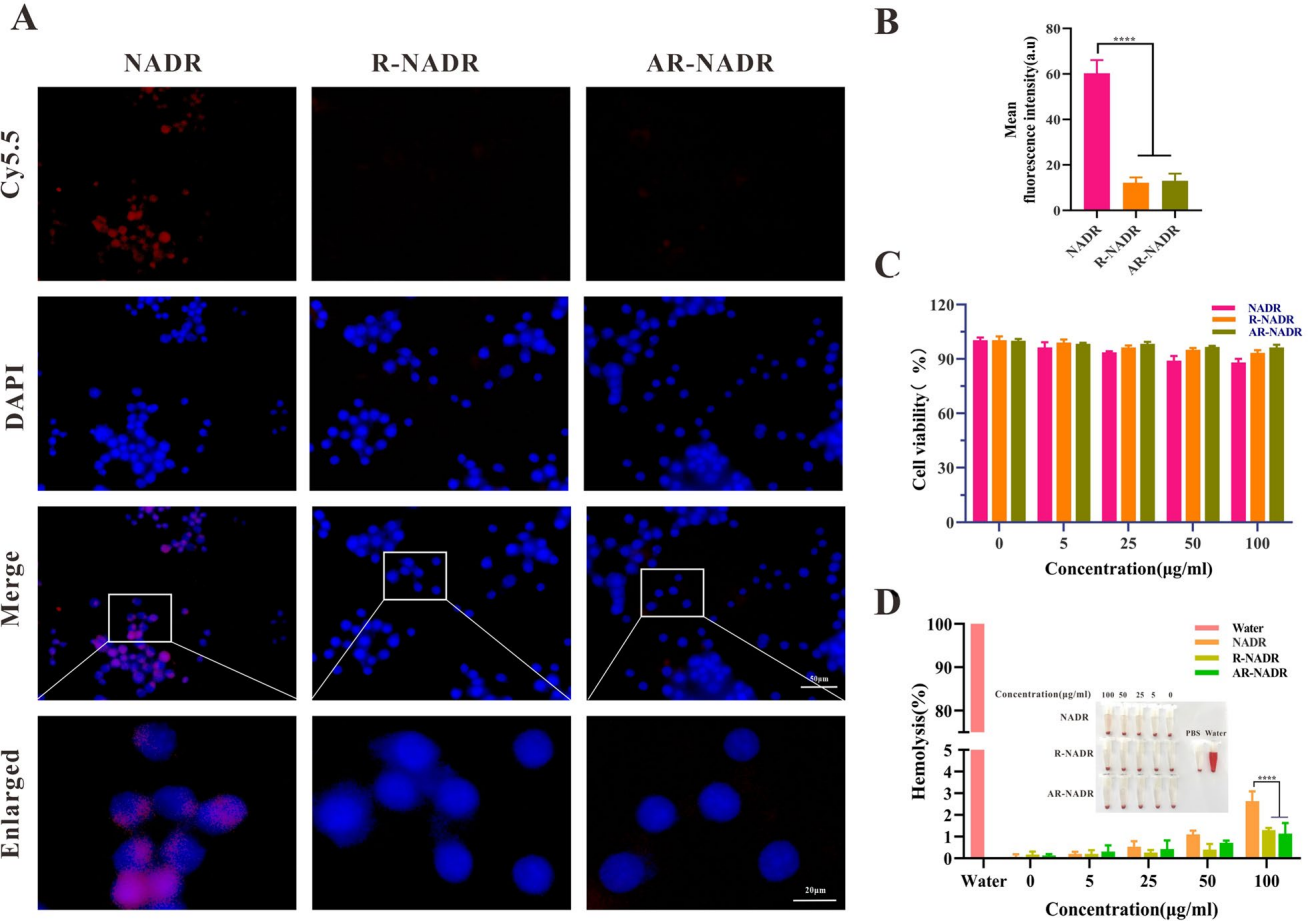
Immune evasion and biocompatibility of AR-NADR in vitro. **A** Fluorescence images of RAW264.7 cells after culture with NADR, R-NADR, and AR-NADR for 6 h. **B** Mean fluorescence intensity of RAW264.7 cells after various treatments for 6 h. **C** The cell viability of HUVECs treated with NADR, R-NADR, and AR-NADR for 24 h. **D** Quantification of hemolysis of RBCs at various concentrations of NADR, R-NADR, and AR-NADR at 37 °C for 2 h. Data are presented as the means ± SD (n = 3) (intergroup comparisons: ****p < 0.0001)

The antiphagocytic effect of AR-NADR in RAW264.7 macrophages was examined to determine the immune escape capacity. As observed in Fig. 5A and B, unlike the R-NADR and AR-NADR treated groups, a mass of red fluorescence accumulated in the RAW264.7 macrophages in the NADR treated group, indicating that after being camouflaged with RBCm vesicles, the immunogenicity of R-NADR and AR-NADR decreased and phagocytosis was significantly inhibited. Consequently, the above characteristics allowed AR-NADR to possess a prolonged circulatory half-life by avoiding recognition and eradication by the reticuloendothelial system in vivo. The results of toxicity assay (Fig. 5C) showed that even at a high dose (100 µg/mL), all preparations had little effect on the cell activity of HUVECs. The results demonstrated that the nanomaterial was nontoxic to normal cells and had good biocompatibility. Additionally, a hemolysis test was performed to confirm that AR-NADR was blood compatible. As indicated in Fig. 5D, R-NADR and AR-NADR hardly caused obvious hemolysis at 2 h (less than 5%). The above findings revealed that AR-NADR possessing good blood compatibility and biocompatibility could be applied for intravenous administration.

### Double target binding ability of AR-NADR

**Fig. 6:**
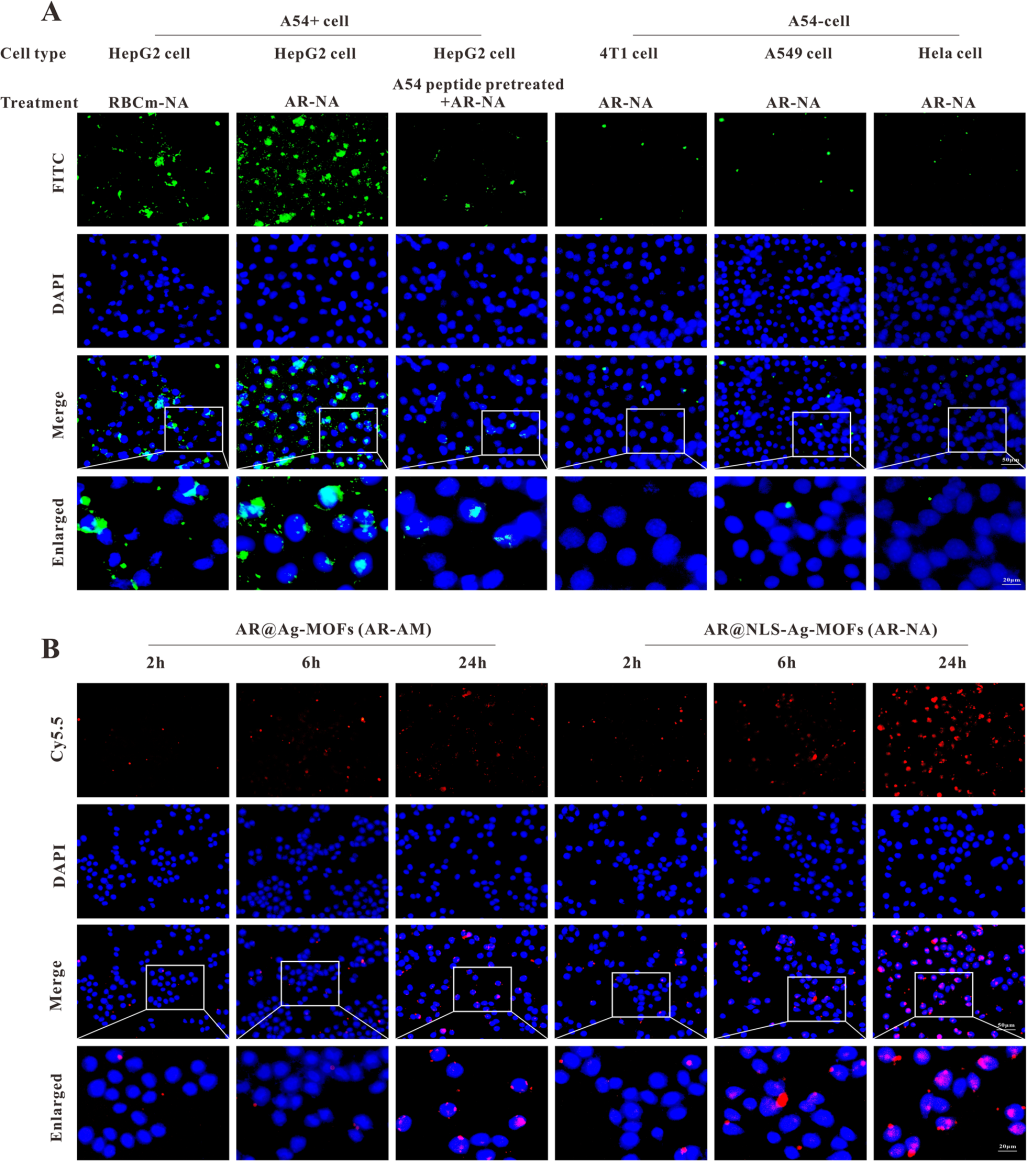
Double target binding ability of AR-NADR. **A** Fluorescence images of HepG2 (A54 + cell), 4T1, A549, and HeLa cells (A54-cell) incubated with various treatments for 12 h. **B** Fluorescence images of HepG2 cellular uptake of AR@Ag-MOFs (AR-AM) and AR@NLS-Ag-MOFs (AR-NA) at 2, 6 and 24 h. Scale bar: 50 μm, 20 μm

As a targeted drug delivery system (DDS), AR-NADR specifically combines with the A54 peptide receptor expressed in HCC cells. The cellular uptake behavior was analyzed to determine its specific binding capability in various tumor cells. The RBCm@NLS-Ag-MOFs (RBCm-NA) and A54-RBCm@NLS-Ag-MOFs (AR-NA) were labeled with FITC expressing green fluorescence. As shown in Fig. 6A, unlike the RBCm-NA treated group, HepG2 cells that highly expressed the A54 peptide receptor on the cell surface (A54 + cells) accumulated abundant green fluorescence in the AR-NA-treated group. Another competition study of the A54 receptor was carried out. The results showed that cells pretreated with free A54 peptide exhibited little intracellular fluorescence even in HepG2 cells. Because the free A54 peptide saturated the A54 receptor on the cell membrane and inhibited the endocytosis of AR-NA mediated by the A54 receptor. The above observations indicated the effect of AR-NA on cell uptake mediated by the A54 receptor. Importantly, minimal green fluorescence was observed in 4T1, A549 or HeLa cells (A54− cells) treated with AR-NA, strongly suggesting that the A54 peptide of AR-NA had the ability to specifically target HCC cells.

To detect the effect of NLS nuclear targeting, the cellular uptake behavior of AR@Ag-MOFs (AR-AM) and AR@NLS-Ag-MOFs (AR-NA) was investigated in HepG2 cells. Cy5.5 was used to label AR-AM and AR-NA so that they displayed red fluorescence. As revealed in Fig. 6B, the level of red fluorescence increased over time in both groups. Notably, the cellular uptake capacity of AR-NA was slightly higher than that of AR-AM. Particularly at 24 h, the AR-NA group expressed a mass of red fluorescence in the cell nucleus, which verified the feasibility of the nuclear targeting of AR-NA after functionalization with the NLS peptide. The cells exhibited enhanced uptake of AR-NA, so the AR-NADR we later constructed could precisely deliver DDP and shRNA to the cell nucleus. The dual-targeting strategy of AR-NADR could specifically target HCC cells while enhancing the cell internalization of nanoparticles, which could effectively transport drugs and shRNA into the cells and improve the antitumor effect.

### Efficient codelivery by AR-NADR of shRNA to suppress NOR1 expression in vitro

**Fig. 7:**
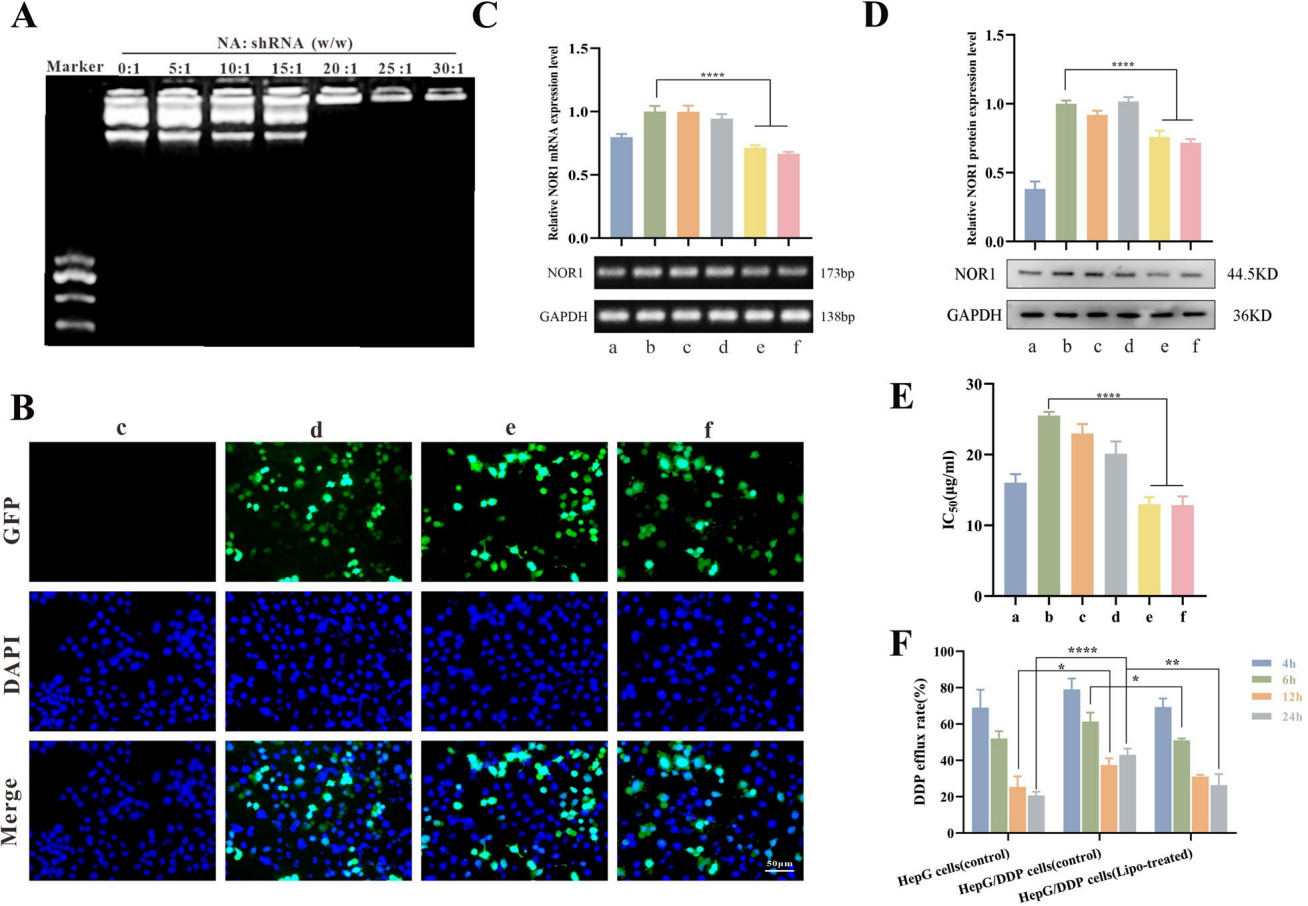
Efficient codelivery by AR-NADR of shRNA to suppress NOR1 expression in vitro. **A** Agarose gel electrophoresis assay of NA/shRNA complexes at different weight ratios. **B** Fluorescence images of GFP fluorescence in different treatments. **C** The detection of *NOR1* mRNA levels by agarose gel electrophoresis assay and **D** NOR1 protein levels by western blot analysis upon different treatments. **E** The IC_50_ values of DDP in HCC cells in different treatment groups. **F** The cell efflux rates of DDP at different time points. (a) HepG2 cells (control), (b) HepG2/DDP cells (control), (c) Free shRNA, (d) Lipo8000-NC (Lipo8000-negative control plasmid), (e) Lipo8000-shRNA, (f) AR-NAR (AR-NA/shRNA). Scale bar: 50 μm. Data are presented as the means ± SD (n = 3) (intergroup comparisons: *p < 0.05, **p < 0.01, ****p < 0.0001)

To investigate the binding capability of NA for NOR1 shRNA, an agarose gel electrophoresis assay was adopted. Different NA/shRNA weight ratios (w/w) were used to prepare NAR, and the movement of shRNA was observed with agarose gel electrophoresis. As shown in Fig. 7A, shRNA binding with NA was gradually impeded when the weight ratio increased from 0:1 to 30:1. When the weight ratios reached or exceeded 20:1, the migration of shRNA was entirely blocked, demonstrating the effective loading of shRNA in NA. An NA/shRNA weight ratio of 20:1 was used to generate AR-NADR to ensure stable and efficient delivery of shRNA.

Furthermore, the in vitro gene transfection efficiency of AR-NADR was evaluated. GFP in the plasmid emitted green fluorescence, which was used to evaluate the transfection efficiency. As shown in Fig. 7B, the level of green fluorescence in the AR-NAR group was analogous to that in the Lipo8000 transfection reagent-treated group (a commercial transfection reagent used as a positive control), indicating successful transfection. Subsequently, the mRNA and protein levels of NOR1 in HepG2 cells and HepG2/DDP cells were measured. As shown in Fig. 7C and D, the agarose gel electrophoresis and Western blot results suggested that compared with HepG2 cells, HepG2/DDP cells overexpressed NOR1. After treatment with Lipo8000-shRNA and AR-NAR, the expression of NOR1 was significantly decreased at both the mRNA and protein levels. As shown in Fig. 7E, HepG2/DDP cells highly expressed NOR1 with a higher IC_50_ value (25.5 ± 0.5 µg/mL) than in HepG2 cells (IC_50_ = 16.04 ± 1.18 µg/mL). NOR1 expression in cells was associated with sensitivity to DDP, and inhibition of NOR1 expression reversed drug resistance in HepG2/DDP cells. The IC_50_ values in the Lipo8000-shRNA and AR-NAR treated groups were 13 ± 0.98 µg/mL and 12.8 ± 1.2 µg/mL, respectively, which were dramatically lower than those in HepG2/DDP cells in the nontreated group. Furthermore, as shown in Fig. 7F, the cell efflux of DDP in HepG2/DDP cells was higher than that in HepG2 cells at 12 and 24 h. When NOR1 expression was inhibited, the cellular efflux of DDP was reduced; as a result, the ingestion of DDP was comparable to that in HepG2 cells. The above results indicated that AR-NAR efficiently delivered NOR1 shRNA. Downregulation of NOR1 reduced the IC_50_ of DDP in HepG2/DDP cells and decreased the efflux of DDP. Thus, AR-NADR presumably reverses chemoresistance by suppressing NOR1expression.

### In vitro antitumor effects of AR-NADR

**Fig. 8:**
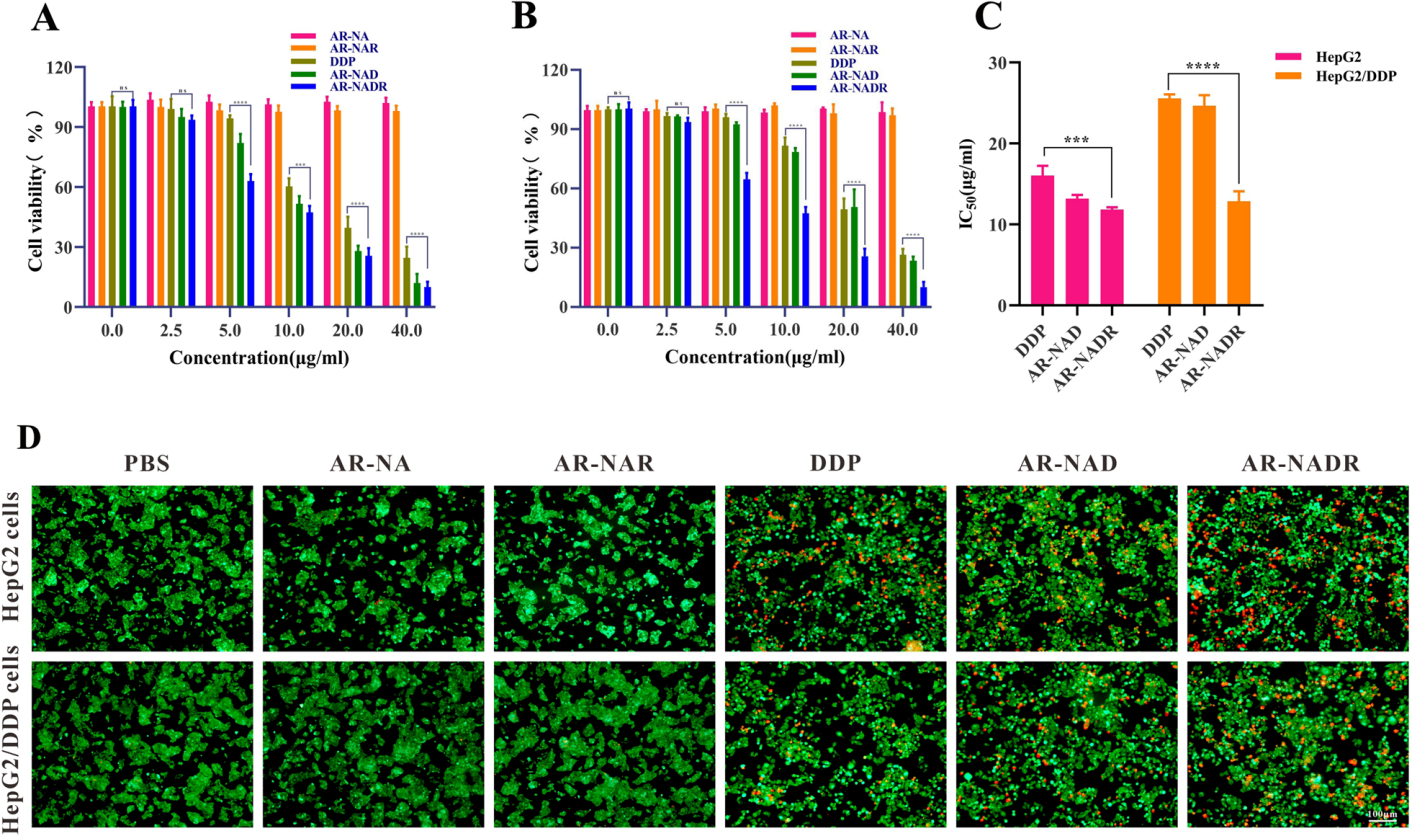
In vitro antitumor effects of AR-NADR. **A** Cell viability of HepG2 cells and **B** HepG2/DDP cells after 24 h treatment with different concentrations of AR-NA, AR-NAR, DDP, AR-NAD and AR-NADR. **C** The IC_50_ values of HepG2 cells and HepG2/DDP cells in the DDP, AR-NAD and AR-NADR treated groups. **D** Live/dead staining of HepG2 cells and HepG2/DDP cells upon various treatments for 6 h. While the living cells displayed green fluorescence, the dead cells exhibited red fluorescence. Scale bar: 100 μm. Data are presented as the means ± SD (n = 3) (ns represents no significance, intergroup comparisons: ***p < 0.001, ****p < 0.0001)

CCK-8 assays and live/dead cell staining were performed to investigate the cytotoxicity of AR-NADR against HCC cells. Compared with the PBS group, the DDP, AR-NAD and AR-NADR groups had significantly inhibited proliferation of HepG2 cells (Fig. 8A) and HepG2/DDP cells (Fig. 8B) in a dose-dependent manner. As shown in Fig. 8C, the IC_50_ value of DDP in HepG2 cells was observably lower than that in HepG2/DDP cells (IC_50_ = 16.04 ± 1.18 µg/mL on HepG2 cells versus IC_50_ = 25.5 ± 0.5 µg/mL on HepG2/DDP cells, denoting > 1.58-fold drug resistance). Moreover, AR-NAD had a stronger HepG2 cell killing effect than DDP alone, with an IC_50_ value of 13.17 ± 0.46 µg/mL. Because of the EPR effect as well as the target function of the nanocarrier, the internalization of DDP drugs by cells was higher [57]. Moreover, the anticancer effect of AR-NADR on HepG2/DDP cells was further improved (IC_50_ = 12.8 ± 1.2 µg/mL), and was almost twofold higher than that of DDP alone. This result further revealed that the effective reduction in the IC_50_ value in the AR-NADR-treated group in HepG2/DDP cells was mainly due to the downregulation of NOR1. Similar to other results (Fig. 5C), the drug-free nanocomposites AR-NA and AR-NAR showed little impact on the viability of HCC cells, indicating the safety of the nanosystem. As shown in Fig. 8D, the staining results of live/dead HepG2 cells and HepG2/DDP cells were in accordance with the results of the CCK-8 assay. The AR-NADR group exhibited apparent red fluorescence (dead cells) in both HepG2 cells and HepG2/DDP cells, indicating that AR-NADR had the most effective antitumor effects in vitro. Most importantly, the AR-NADR we constructed could potentially be an effective gene delivery vector for reversing drug resistance with powerful antitumor effects in HCC.

### Mechanisms of AR-NADR against drug-resistant HCC cells

**Fig. 9:**
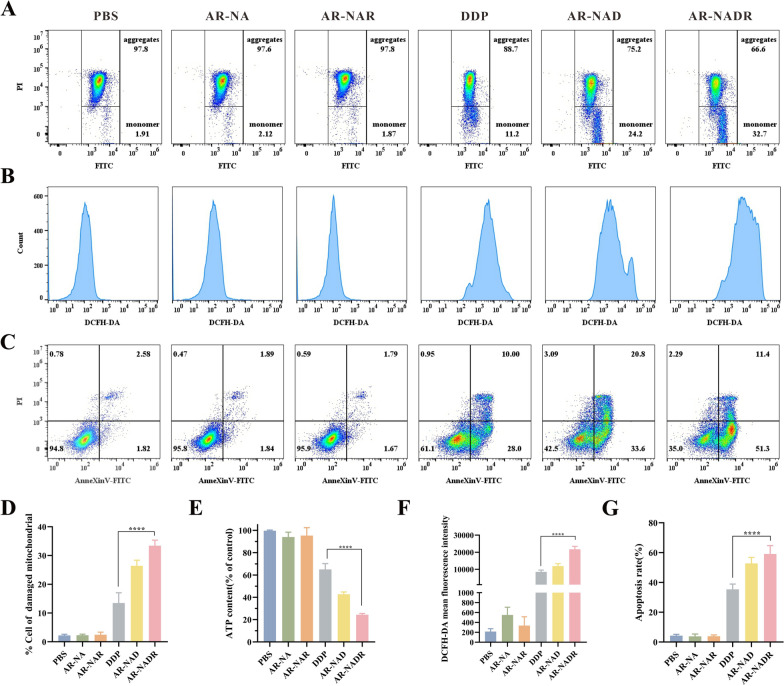
Mechanisms of AR-NADR against drug-resistant HCC cells. **A** Representative flow cytometric analysis of JC-1 staining to detect MMP depolarization in HepG2/DDP cells treated with PBS, AR-NA, AR-NAR, DDP, AR-NAD, and AR-NADR for 12 h. **B** The production of ROS was detected by DCFH-DA in HepG2/DDP cells upon different treatments for 12 h. **C** Representative flow cytometric analysis of AnneXinV-FITC/PI-stained HepG2/DDP cells after incubation with different treatments for 12 h. **D** The proportion of HepG2/DDP cells in the lower quadrant (representing damaged mitochondrial cells). **E** Effects of different treatments on the intracellular ATP contents of HepG2/DDP cells. **F** The DCFH-DA mean fluorescence intensity after various treatments of HepG2/DDP cells. **G** The apoptosis rate of HepG2/DDP cells upon indicated treatments. Data are presented as the means ± SD (n = 3) (intergroup comparisons: ****p < 0.0001)

To thoroughly investigate the mechanism of the anticancer efficacy of AR-NADR against DDP-resistant cells, we sequentially investigated the MMP, ATP, ROS and apoptosis of HepG2/DDP cells. Mitochondrial injury characterized by decreased MMP is one of the hallmark events that occurs during the early stage of apoptosis. The change in MMP after apoptosis induction in cells allows the membrane permeability to be altered [58]. As shown in Fig. 9A, the cells were divided into upper quadrants (aggregates) and lower quadrants (monomers), indicating normal or damaged mitochondria of cells, respectively. The proportion of cells in the lower quadrant treated with AR-NADR was 33.4 ± 1.9%, which was higher than that treated with DDP (13.4 ± 3.5%) (Fig. 9D). Intracellular ATP production is affected if mitochondrial functionality is impaired [59]. Hence, compared with the DDP-treated group, the AR-NADR treated group showed significantly lower ATP content (Fig. 9E). The above results suggested that the AR-NADR-treated group exhibited more severe damage to mitochondrial function. Furthermore, overproduction of ROS is involved in damaging cellular components such as DNA, proteins and lipids, leading to apoptosis [60]. AR-NADR caused an obvious rightward shift of the peak, explaining it could induce more ROS production than other groups (Fig. 9B and F). In addition, as shown in Fig. 9C and G, the apoptotic rate of the AR-NADR-treated group was 59 ± 5.5%, which was higher than the group treated with DDP alone (35.3 ± 3.5%), revealing that AR-NADR induced more cell apoptosis. The AR-NA and AR-NAR treatments had little effect on the apoptotic rates. In conclusion, AR-NADR could accelerate mitochondrial injury and cell apoptosis and promote high ROS and ATP levels, which caused more cell death.

### Biodistribution of AR-NADR in vivo

**Fig. 10:**
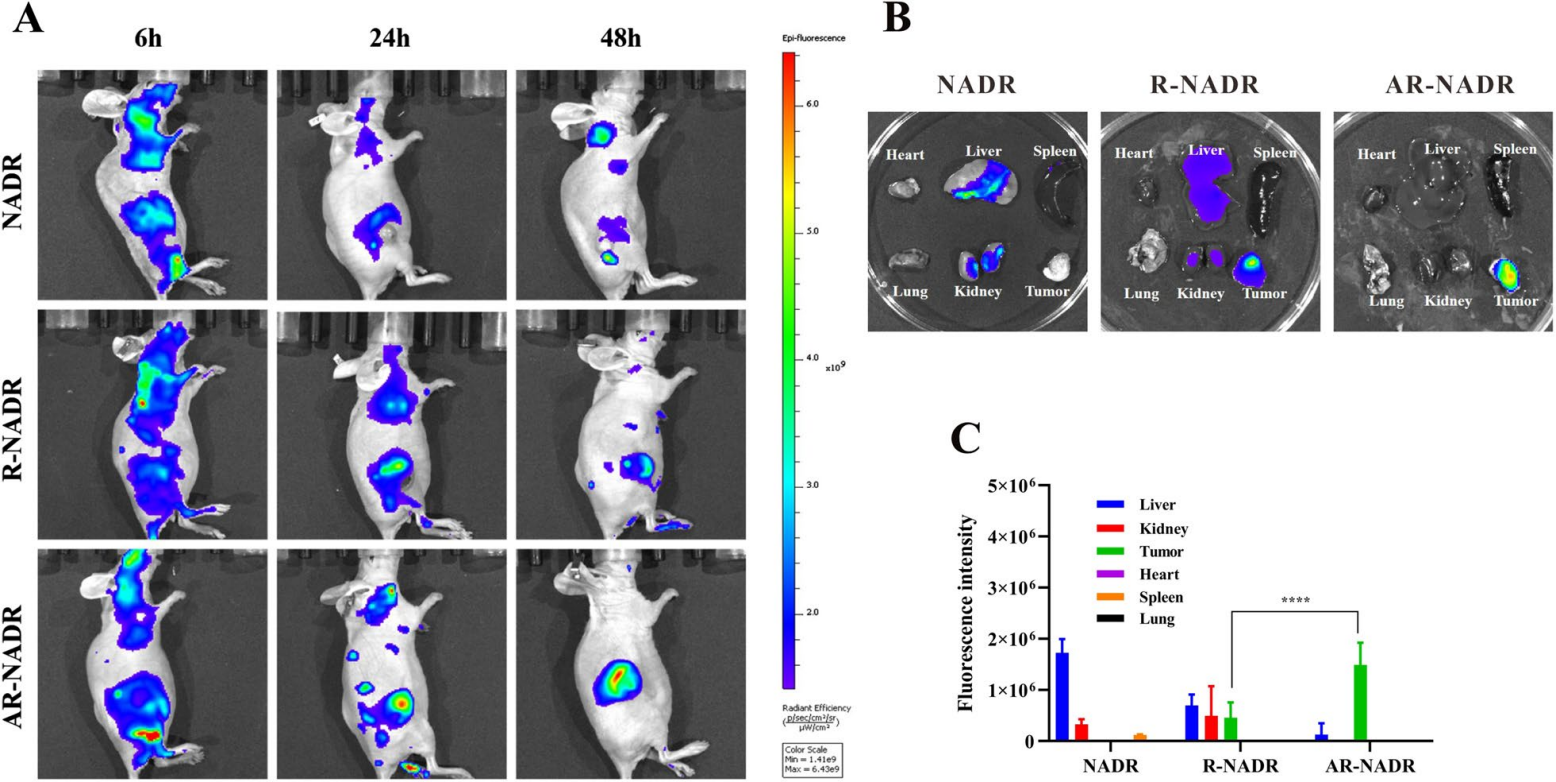
Biodistribution of AR-NADR in vivo. **A** Fluorescence images of mice in vivo after intravenous treatments with NADR, R-NADR, and AR-NADR at 6, 24, and 48 h. **B** Fluorescence images of the main organs (heart, liver, spleen, lung, and kidney) and tumors after treatment with NADR, R-NADR, and AR-NADR for 48 h. **C** Semiquantitative assessment of fluorescence signal in main organs and tumors at 48 h. Data are presented as the means ± SD (n = 3) (intergroup comparisons: ****p < 0.0001)

The efficiency of drug accumulation in tumors is a significant element that needs to be considered in drug delivery systems. To investigate the tumor targeting capability of AR-NADR in vivo, its biodistribution in a tumor-bearing mouse model was evaluated. As indicated in Fig. 10A, little fluorescence signal reached the tumor site in the NADR-treated group. Due to the EPR effect and immune escape, the R-NADR-treated group showed limited of fluorescence signal in the tumor site at 24 h post-injection. More importantly, the fluorescence signal increased obviously at 24 h in the tumor site of the AR-NADR group due to the targeting effect of the A54 peptide. Ex vivo images of major organs (heart, liver, spleen, lung, and kidney) and tumor tissues isolated from the sacrificed mice at 48 h post-injection were obtained (Fig. 10B and C). The results showed that, the fluorescence accumulated strongly at the tumor site in the AR-NADR-treated group and rarely accumulated in other organs. These outcomes confirmed that the immune escape of AR-NADR prolonged its time in the blood circulation and achieved efficient aggregation at the tumor site in response to the EPR effect and A54 peptide targeting [61].

### In vivo antitumor effects of AR-NADR

**Fig. 11:**
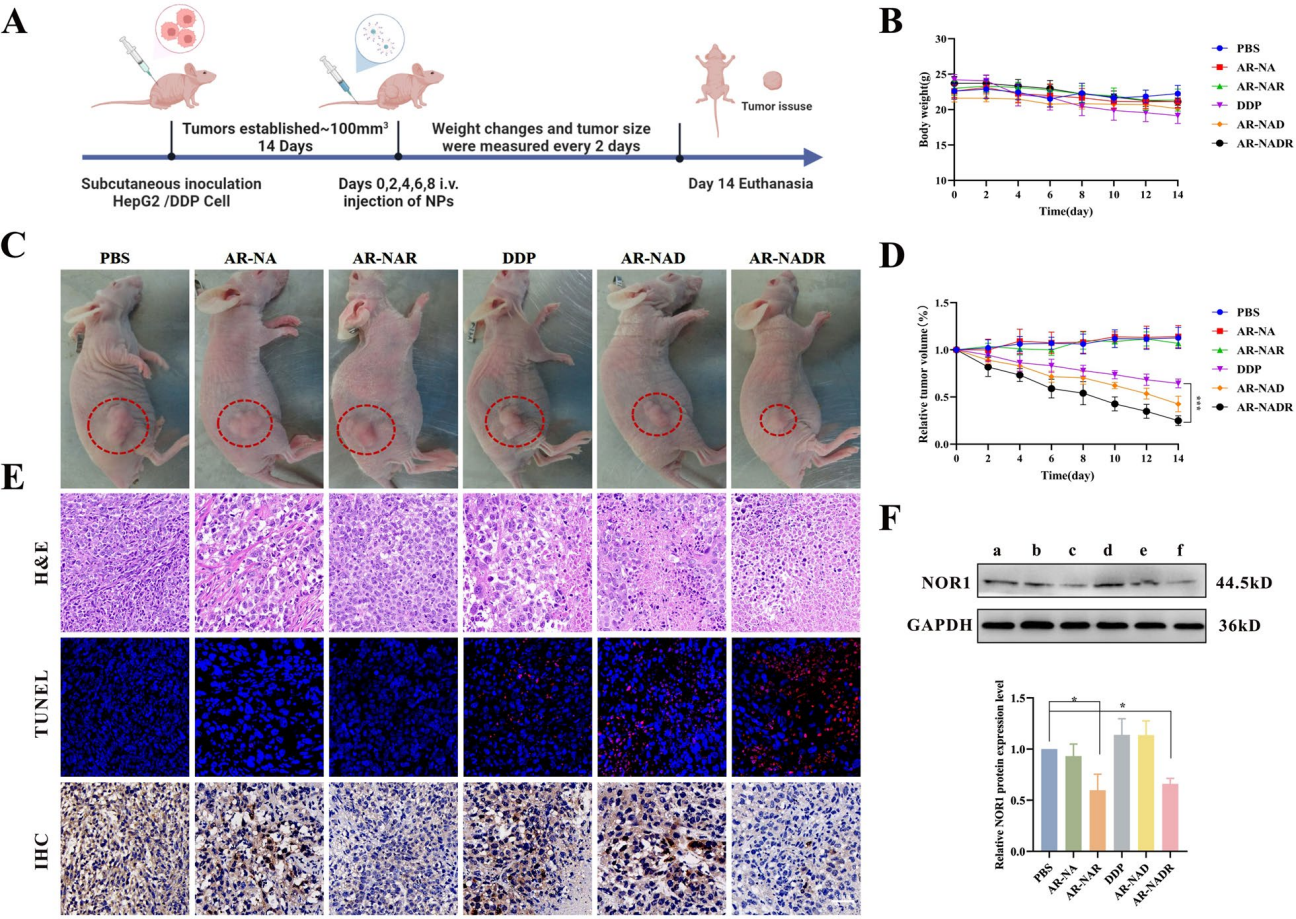
In vivo antitumor effects of AR-NADR. **A** Treatment regimen. **B** Body weight of mice after treatment with PBS, AR-NA, AR-NAR, DDP, AR-NAD, and AR-NADR. **C** Photographs of representative animals in each group. **D** Changes in tumor volume after various treatments. Tumor volumes were normalized to baseline values. **E** (a) H&E staining, (b)TUNEL staining and (c) NOR1 IHC of tumor tissues at 14 days after injection with various treatments. **F** NOR1 protein levels by Western blot analysis upon different treatments. Semiquantitative assessment of Western blot bands. (a) PBS, (b) AR-NA, (c) AR-NAR, (d) DDP, (e) AR-NAD, and (f) AR-NADR. Scale bar: 50 μm. Data are presented as the means ± SD (n = 3) (Intergroup comparisons: *p < 0.05, ***p < 0.001)

The therapeutic efficacy of AR-NADR in vivo was examined in subcutaneous HepG2/DDP cell tumor-bearing nude mice (Fig. 11A). The tumor-bearing mice were randomly divided into six groups (n = 5 per group) and then intravenously injected with PBS, AR-NA, AR-NAR, DDP, AR-NAD, or AR-NADR 5 times (2 mg/ml DDP, every 2 days). The tumor size and body weight of the mice were measured every second day. As shown in Fig. 11Β, the mouse body weight displayed little change in each group except in the DDP-treated group, which exhibited certain side effects (weight loss of 20 ± 3.3%). However, the AR-NADR group showed markedly inhibited tumor growth (the inhibition rate was 75 ± 5%) on the 14th day compared with the other treatment groups (Fig. 11C and D). This finding demonstrated that the anticancer activity of AR-NADR could be markedly enhanced because of the long half-life, targeting effects and inhibition of NOR1 expression. At 14 days, all the mice were euthanized, and H&E staining, TUNEL assays and IHC of tumor tissues were performed. The results showed that the tumor tissues of the AR-NADR-treated group displayed obvious nuclear condensation and fragmentation, while the apoptotic fluorescence intensity was notably enhanced, unlike the staining results of samples from the other groups (Fig. 11E). Moreover, to verify the transfection efficiency of AR-NADR in vivo, IHC and Western blotting were also performed to analyze NOR1 expression in tumor tissues. As shown in Fig. 11E, the results of IHC staining revealed that the number of NOR1-positive cells (brownish yellow) was the lowest in the AR-NAR and AR-NADR groups, indicating that the nanosystem we constructed could effectively reduce NOR1 expression. Western blotting outcomes further proved the expression level of NOR1 protein in tumor tissues (Fig. 11F). Based on the above results, it was firmly believed that the Ag-MOF-assisted assembly strategy to accomplish effective coencapsulation of drugs and shRNA was feasible. Taken together, these results show that AR-NADR can improve anticancer activity, which provides a multiple-strategy treatment model with the potential for clinical translation.

### Biocompatibility of AR-NADR in vivo

**Fig. 12:**
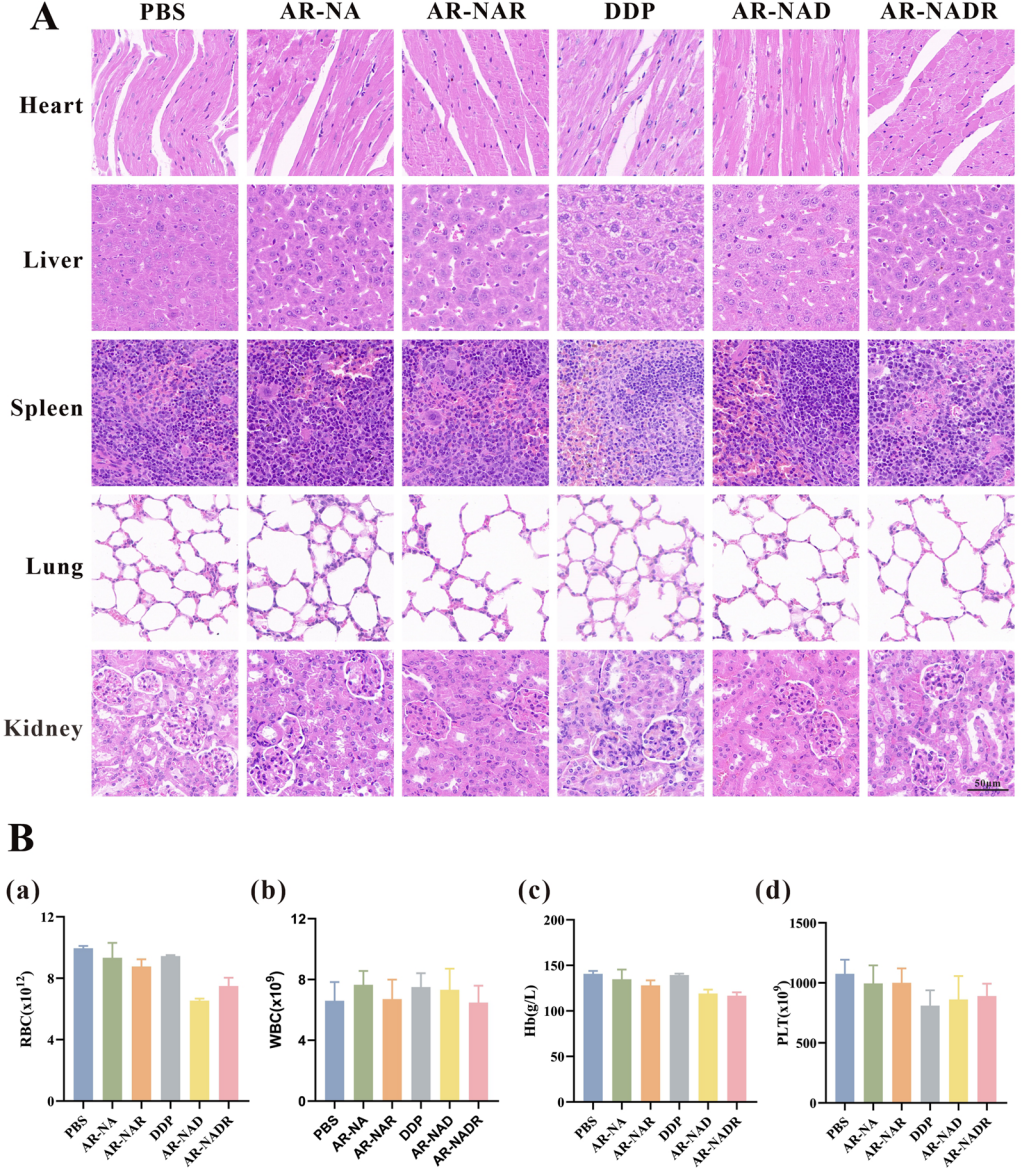
The biocompatibility of AR-NADR in vivo. **A** Representative H&E-stained images of the major organs (heart, liver, spleen, lung, and kidney) harvested from mice at 14 days after various treatments (PBS, AR-NA, AR-NAR, DDP, AR-NAD, and AR-NADR). **B** Values of hematological parameters assayed upon various treatments. (a) RBC, (b) WBC, (c) Hb and (d) PLT. Scale bar: 50 μm. Data are presented as the means ± SD (n = 3)

The biocompatibility of nanomaterials is attracting increasing attention for addressing medical and biological problems [62]. In this study, the safety profiles of AR-NADR in vivo were investigated. As shown in Fig. 12A, H&E staining of the major organs (heart, liver, spleen, lung, and kidney) displayed negligible histomorphological changes. In addition, similar to the PBS treatment group, there were no distinct alterations in hematological indices (RBC, WBC, PLT, and Hb) after injections of AR-NA, AR-NAR, DDP, AR-NAD, or AR-NADR (Fig. 12B). The body weight of mice also showed no obvious change (Fig. 12B). The above findings demonstrated that AR-NADR possessed lower toxicity, fewer side effects and good biocompatibility.

## Conclusion

In this research, we revealed the potential relationship between NOR1 and DDP-resistant HCC. We successfully constructed a novel nanosystem, namely, AR-NADR, which contained A54-modified RBC membrane vesicles as the shell and an NLS modified Ag-MOF loaded with DDP and NOR1 shRNA as the core for the treatment of cisplatin resistance in HCC. This drug delivery system possesses many advantages, such as double targeting, satisfactory biocompatibility, and release properties in response to weak acidic environmental stimuli, effective gene transfection and synergistic antitumor effects. Consequently, AR-NADR is regarded as a reversal agent providing extraordinary potential for overcoming cisplatin resistance in tumors via multiple mechanisms. AR-NADR not only offers an effective strategy for the clinical application of gene therapy but also unveils a novel approach with applications in the biomedical field.

## Data Availability

All data generated or analyzed during this study are included in this published article.
